# *Mind the Gut*—displaying microbiome research through artistic collaboration

**DOI:** 10.1080/16512235.2018.1555433

**Published:** 2018-12-25

**Authors:** Adam Bencard, Louise Emma Whiteley

**Affiliations:** Medical Museion, Institute of Public Health and Novo Nordisk Foundation Center for Basic Metabolic Research, University of Copenhagen, Copenhagen, Denmark

**Keywords:** Exhibitions, science communication, medical humanities, history and philosophy, art and science

## Abstract

This paper presents the *Mind the Gut* exhibition, opened in 2017 at the Medical Museion, the University of Copenhagen's museum for the culture and history of medicine. It is an experimental exhibition combining science, art, and history in an examination of the relationship between mind and gut, including the trillions of microbes that inhabits them. *Mind the Gut* was the result of a 2-year-long research and curatorial process, which began in 2015 when Museion was awarded the Bikuben Foundation Vision Award. The exhibition brings together the long history of attempts to understand and intervene in the relationship between mind and gut, between emotions and digestion with cutting-edge biomedical research, and includes the perspectives of science, medicine, and personal experience, via a combination of artworks, historical objects from the Medical Museion collections, items from laboratories, and individual stories. The exhibition is organized around different ways the body has been handled in order to intervene in interactions between mind, gut, and bacteria, including imaging, electrifying, feeding, drugging, and opening surgically. This paper outlines some of the thoughts on science communication that motivated the exhibition, discussing why the displays emphasize the exploratory over the explanatory. Also discussed are several artistic collaborations that formed part of the displays. Ultimately, *Mind the Gut* is created to be a public space that encourages reflection and curiosity, by showing how biomedicine fits into social, cultural, historical, and directly personal contexts. The exhibition does not aim to provide answers about what food the visitors should eat or what the truth of how gut and brain interactions might be. Rather, it emphasizes process over result, hopefully encouraging the visitors to ask their own questions of the relationship between mind and gut, between body and microbes.

In October 2017, the *Mind the Gut* exhibition opened at Medical Museion, offering glimpses into the strange history of our attempts to understand and treat the relationship between brain and belly (see Figure 1, Figure 2). Medical Museion is the University of Copenhagen’s museum for the culture and history of medicine, integrated with an interdisciplinary research group and housing the ‘Metabolism in Culture’ program of the Novo Nordisk Foundation Center for Basic Metabolic Research.

**Figure 1.**
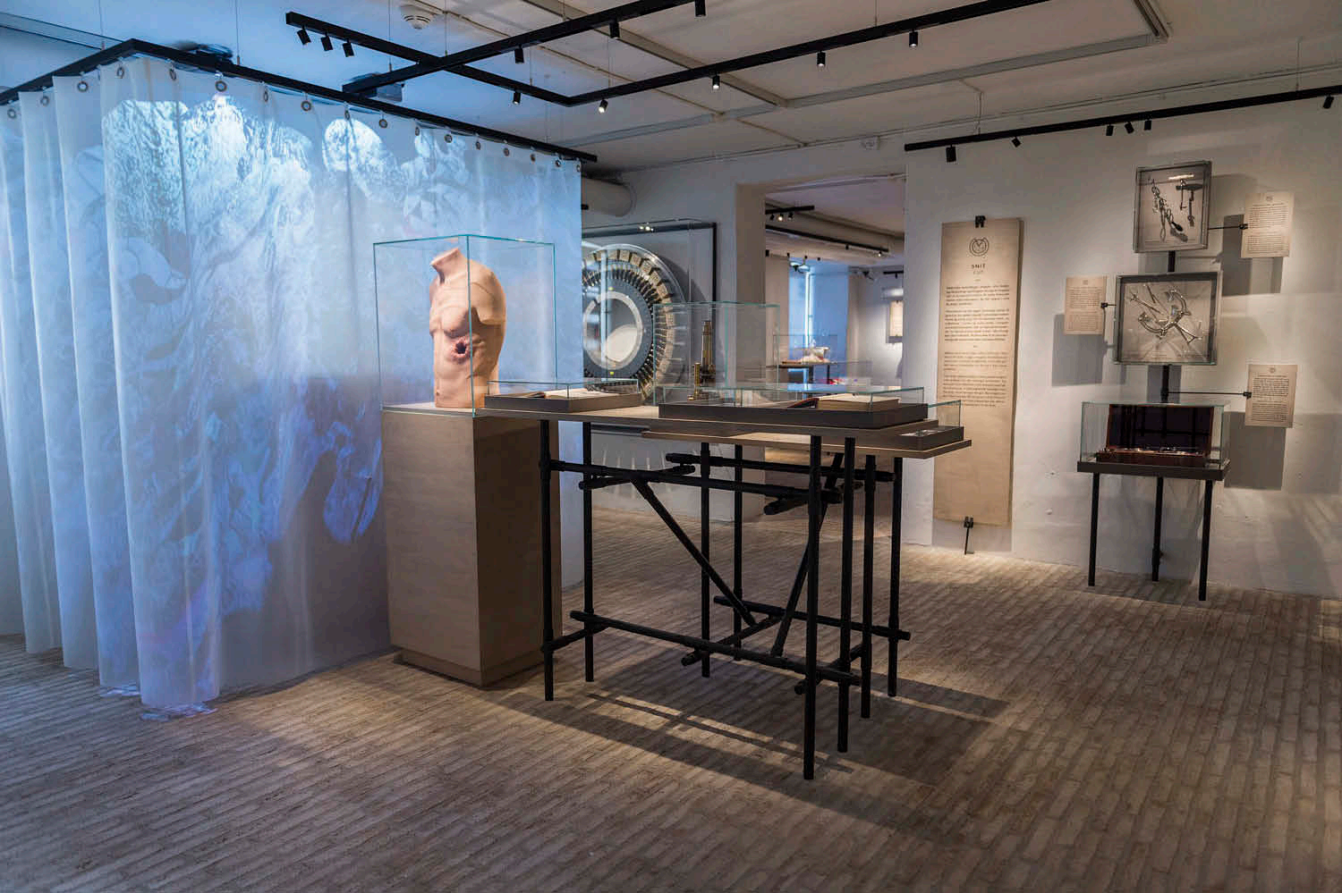
View from inside the *Mind the Gut* exhibition. The exhibition is located in the basement of the Medical Museion, a space filled with wiring and ventilation. These spatial qualities resonate with the themes of complexity and connectedness at the heart of the exhibition, and are also echoed in the exhibition design. Copyright Medical Museion.

**Figure 2.**
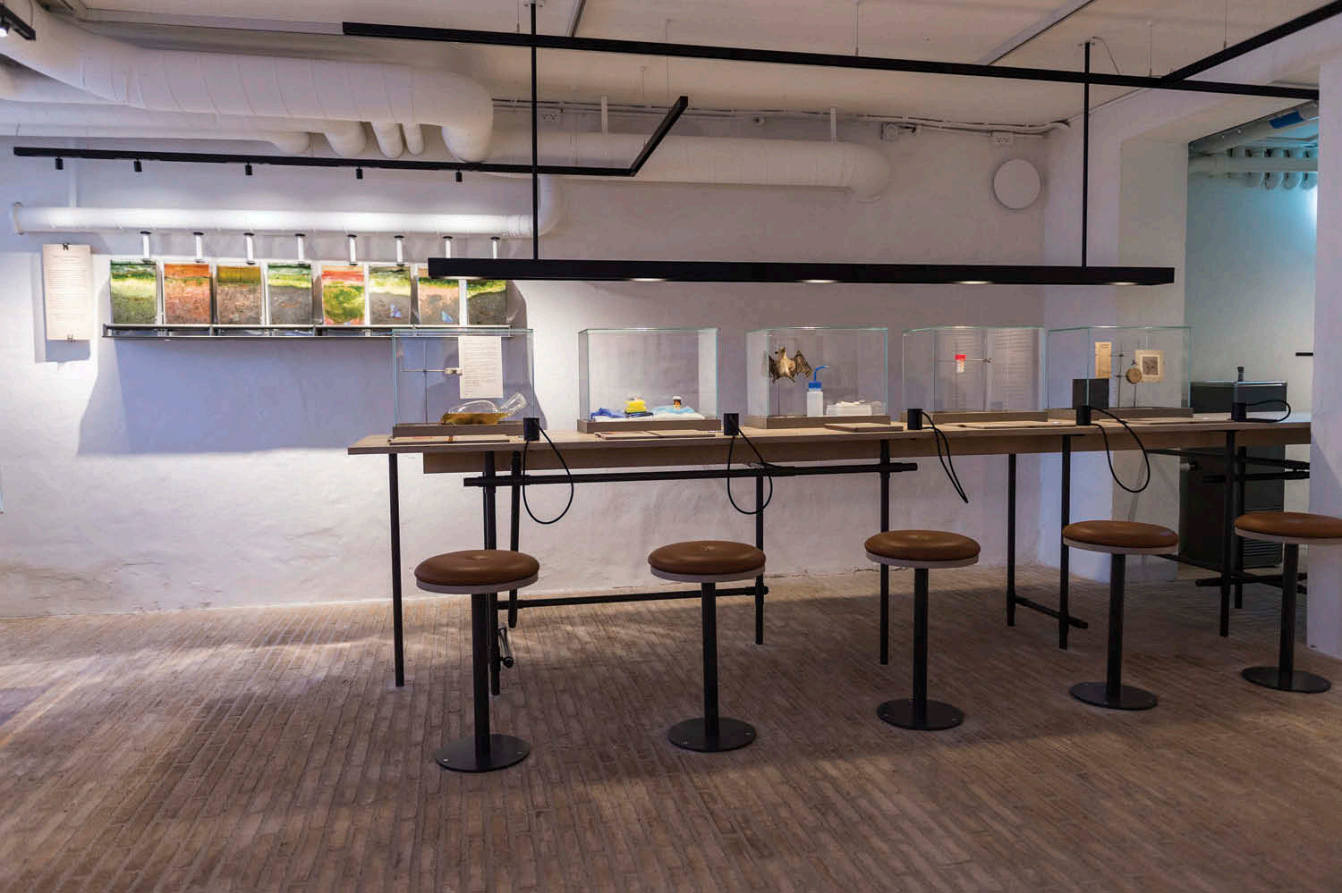
*Mind the Gut* is organized in a series of themes, each showing a type of view upon or intervention in the relationship between mind and gut. Seen here is the theme ‘cultivate’, which features five people who in various ways have tried to manipulate their gut microbes. In the back are a series of Winogradsky columns, living microbial ecosystems with distinct aesthetic qualities.

**Figure 3.**
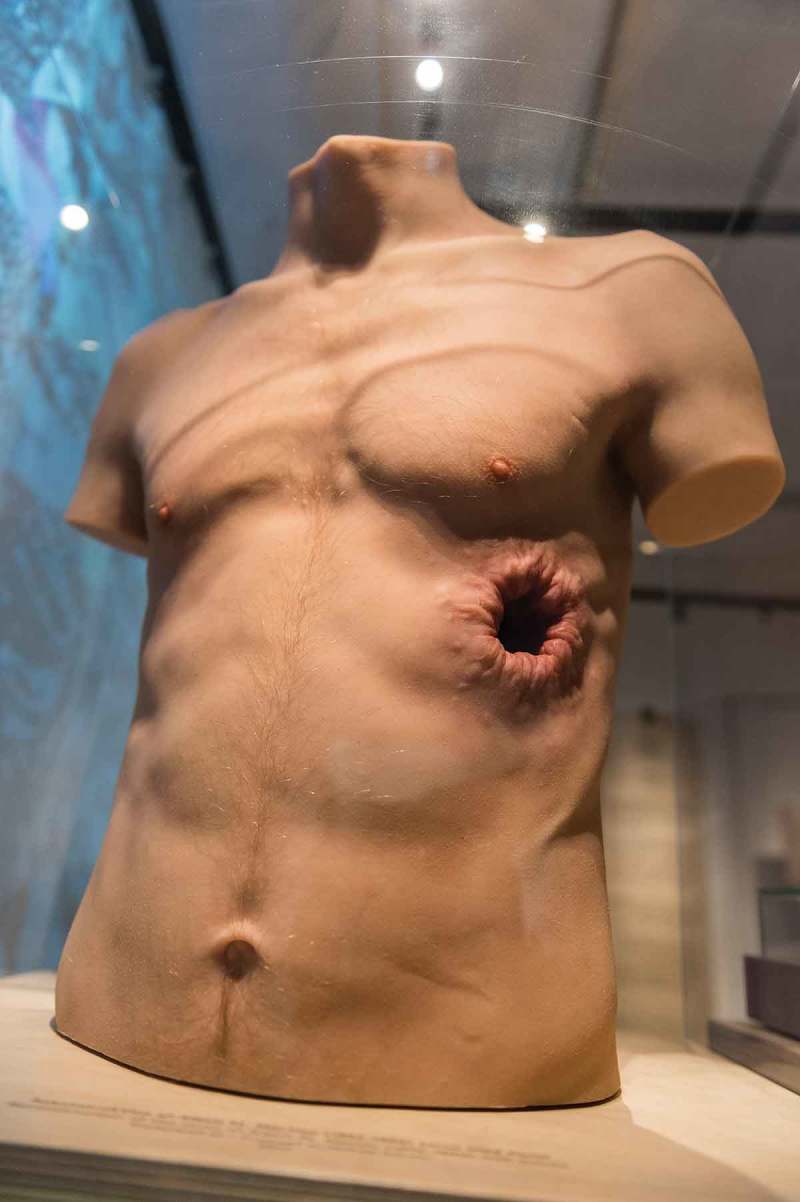
Silicone torso made to resemble Alexis St. Martin, a young man who in 1822 survived a gunshot wound to the stomach but developed a permanent fistula. He was treated by the physician William Beaumont, who later experimented on him by tying food on silk strings and inserting them into the stomach through the fistula. Beaumont’s pivotal observations was published in 1838, in the book Experiments and Observations on the Gastric Juice, and the Physiology of Digestion. Copyright Medical Museion.

**Figure 4.**
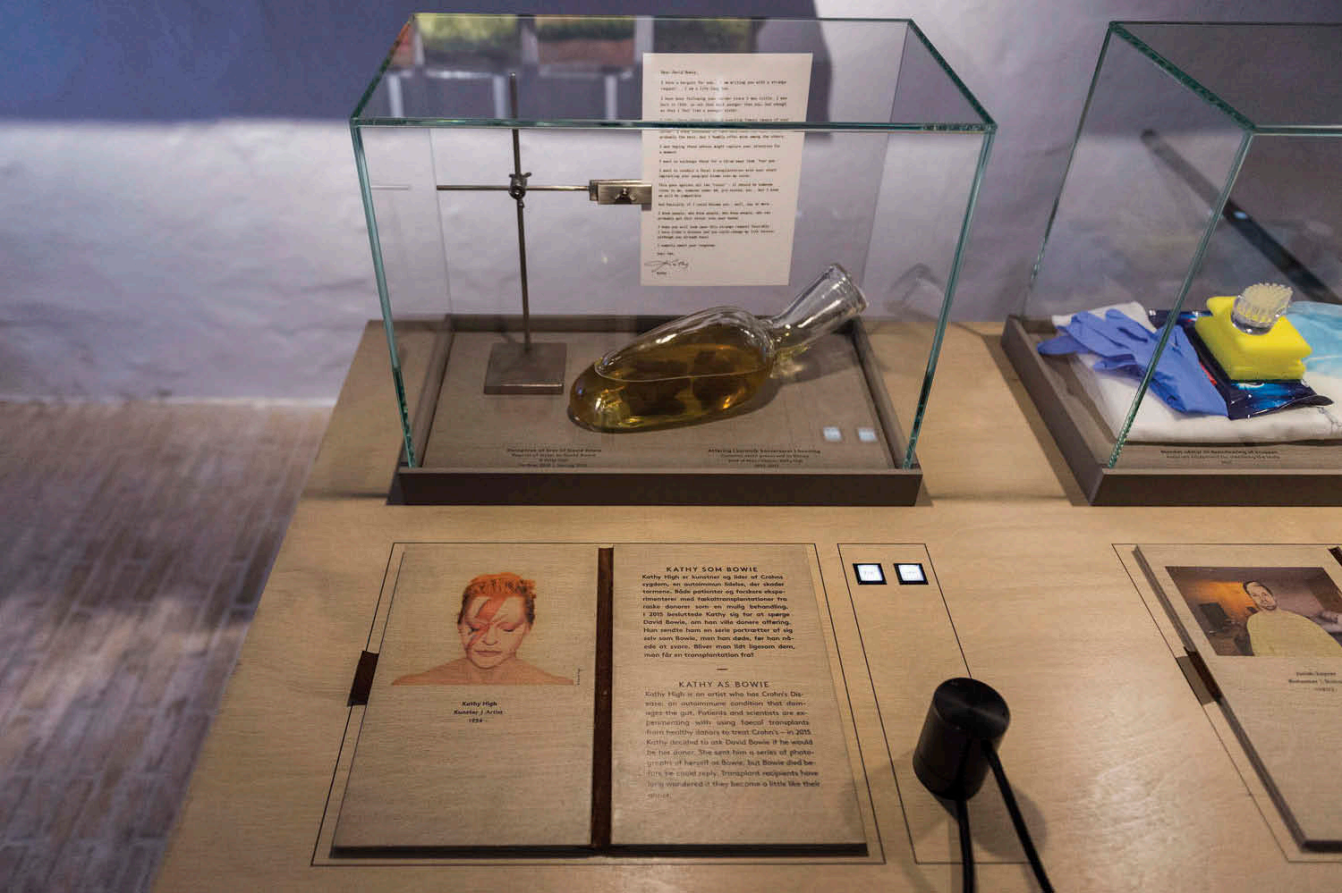
The work of artist Kathy High as featured in the exhibition. In the display case is a copy of the letter she wrote to David Bowie, as well as a glass vessel containing ceramic stool in honey, from her series The Bank of Abject Objects. The audience can also listen to an interview with High about her work. Copyright Medical Museion.

*Mind the Gut* was the result of a 2-year-long experimental research and curatorial process, which began in 2015 when Museion was awarded the Bikuben Foundation Vision Award. The Vision Award is, unlike most exhibition awards, not given retrospectively to an already completed exhibition, but is given to a promising and experimental exhibition *concept*. The winning institution is granted 3 mill DKK to realize the concept, turning it from an idea into a finished exhibition. The time and means provided by the award allowed us to engage in a properly experimental curation process, and an exhibition that cut across science, art, and history. This paper outlines some of the ideas that went into its making, including thoughts on science communication and the relationship between art and science, with a focus on the unique challenges presented by attempts to display the microbial realm and its relationship to our human-sized experience.

As the title suggests, *Mind the Gut* examines the relationship between mind and gut, between brain and bowels including the trillions of microbes that inhabit them. From a public engagement and museum perspective, this was in some ways an easy choice; it is a ‘hot topic’ and an interesting case study of a complex, unsettled research field with potentially profound implications for both medicine and culture. Yet gut-brain-microbiome (GBM) interaction is a complex relationship, as difficult to display as it is to study; contemporary scientific research struggles to disentangle inputs and outputs, conditions and effects. As we discovered, some of our contemporary concerns and scientific challenges have a long history. Doctors and scientists have long been intrigued by both local questions of disease and discontent, and the implications for how we understand the human organism. And throughout history people have worked on mind-gut connections in their daily lives, changing what they eat, how they sleep and exercise, taking supplements, fermenting foods, having enemas, purchasing commercial brain stimulation kits, and so on. The exhibition brings together this history with cutting edge research, and brings together the perspectives of science, medicine, and personal experience, via a combination of artworks, historical objects from the Medical Museion collections, items from laboratories, and individual stories. The exhibition is organized around different ways the body has been handled in order to intervene in interactions between mind, gut, and bacteria, including imaging, electrifying, feeding, drugging, and opening surgically.

Microbiome-gut-brain (MGB) research promises to have profound implications for a number of the pathologies and health problems that characterize our post-industrial societies—and while these promises are yet to be fulfilled, public culture and medical practice are already responding to their possible implications. It is thus a tempting case for anyone interested in science-society relations—they are evolving fast and in public. Fermentation workshops and other bacterially driven food practices are everywhere, DIY fecal transplant videos can be found online, open science projects are selling personal microbiome sampling kits, probiotics are becoming more mainstream, there are a steady stream of TED talks and media reports, best-selling science journalism books, microbiome cookbooks, microbially based makeup products, and much more. Alongside the more practical health implications of mind-gut-microbiome research, it also seems to offer a fundamentally different perspective on long-held views on human development (Pradeu [1], Gilbert [2]), our sense of self (Hird [3], Bencard [4]), and our connection to our environments (McFall-Ngai et al. [5]). It engages a complex, environmentally entangled body, whose very existence is interwoven with nonhuman life—can we therefore talk of our bodies as simply human? How should we conceptualize the kinds of relationship we have to the microscopic organisms that live on and in us? Does our emotional state belong solely to us? Thus we engaged the research as a study of how science and culture is interwoven and plays into deeply individual and existentially resonant experiences. All this makes for a profoundly messy and entangled field, and a rich and engaging topic for on-going experiments at the museum in science communication and public engagement.

## An interdisciplinary process for an interdisciplinary topic

The concept behind *Mind the Gut* was built on a merger of an experimental content—the rapidly developing research field of MGB interaction—with an experimental form: an open-ended cocuratorial process which involved scientists and artists from the start, as well as historical curators. In other words, we wanted to see what would happen if we invited scientists and artists to be part of handling the ‘total medium’ of the exhibition, rather than just using scientists as sources or simply commissioning artists to produce works based on the exhibition themes. We wanted to make them part of a shared discussion and a longer-term process, aimed at breaking down disciplinary boundaries. We wanted to avoid making an exhibition that had a science section, an art section, and a history section, but rather look for questions that arose at the overlaps of—or even out of interactions between—those practices. This was in part a reaction to more traditional approaches to involving artists and scientists in museums of science, technology, engineering, and medicine, which can tend to instrumentalise their expertise toward an (often unnegotiated) communicative goal (e.g., Born and Berry [6]).

We started with an open call for collaborators, in which we asked for ‘four curious, inventive collaborators interested in crossing disciplinary boundaries to join our team’. Successful applicants were expected to join 12 workshops over an 18-month period, as well as participating in a 2-day international conference following the conclusion of the project; they would contribute to the exhibition content in a manner to be agreed during the process; and participate in three research interviews about the project conducted during and after the process. We received 155 applications from a wide range of people—artists, scientists, chefs, philosophers, art curators, cultural historians, speculative designers, and science communicators. With the help of a jury, we ended up choosing five cocurators; three artists; and two scientists. The team then embarked on the year-long journey until the exhibition was opened to the public. Part of the reasoning behind this process was to create data for our own academic research on cocuration and transdisciplinary collaboration; in essence, embedding a research project within the exhibition project.

In setting up this process, we were inspired by scholars Dez Fitzgerald and Felicity Callard, and their work as part of the first interdisciplinary residency at The Hub at Wellcome Collection in London. Their project was entitled Hubbub, and was dedicated to exploring the dynamics of rest, noise, and work. It consisted of a 50-strong international collective of social scientists, artists, humanities researchers, scientists, broadcasters, public engagement professionals, and mental health experts. Through this experimental program, Fitzgerald and Callard developed the concept of ‘experimental entanglements’ as a way to go beyond traditional disciplinary boundaries, and as a way of creating what they call an awkward intradisciplinarity—awkward because there is no set structure, and intra- rather than inter-, because it is set up to go beyond an exchange between different disciplines and hopefully become a process in which the members of the working group impact each other and the object studied and produced. They argue that this approach is a way, as they write, to ‘to help scholars circumvent a burgeoning, but bloodless and sterile, literature on “interdisciplinarity” between the social sciences and the life sciences’ (Callard and Fitzgerald [7])

Applying an experimental, cocuratorial approach to produce an exhibition about biomedical science also built on a decade of museological research and practical experimentation at Medical Museion, which has grasped the challenge of communicating contemporary biomedical research, which can often be complex, opaque, and intangible—not the natural choice for museums that like to exhibit medium-sized, easily interpretable objects (see Söderqvist et al. [8], Whiteley et al. [9]). *Mind the Gut* represented another step in this journey, via an unusually lengthy and open process that aimed to predetermine as little as possible about the roles each participant should play. *Mind the Gut* was thus set up as a science communication experiment in how to use the exhibition medium to display, investigate, and invite audiences to engage in GBM interaction research *as process*, and driven by the hypothesis that ‘experimentally entangled’ cocuration might help us to do so.

Our commitment was to showing science *in* and *as* process, and as culturally embedded, relevant, and resonant, leads to an emphasis on the exploratory over the explanatory. In other words, we wanted to show science as an on-going and open-ended exploration, rather than a progressive fact-based process of explanation. For example, we followed three scientific research projects, two of which were still ongoing at the time, not to put the results on display but rather to show what they look like in practice. We filmed the day-to-day work of the scientists, we collected equipment and animal specimens used in the research, and we worked with the scientists to create publicly digestible diagrams of their experimental setups and hypotheses. The exhibition emphasizes the detective-like aspect of this work, portraying it as something at once incredibly detailed, sophisticated and high tech, as well as open, intuitive, and occasionally downright strange. There has been a general tendency in museums of science to push against traditional modes of only representing science as a slow, steady, objective march toward truth; instead, its open-ended nature is emphasized, communicating about the processual nature of scientific work as much as its results. With *Mind the Gut* this felt particularly pertinent, due to the unsettled nature of GBM interaction research—overselling results and making overly strong claims about causal relationships between microbes, moods, and mental states is a real danger.

## Art and the microbiome

An important aspect of the cocuration approach to exhibiting complex science as process was collaboration with artists. In recent decades, it has been increasingly popular for scientific institutions and science museums to collaborate with artists. Regina Born and Mathew Berry categorized art-science collaboration as being driven by logics of *accountability, innovation,* or *ontology*. The logic of accountability is perhaps the most well-known and practiced. Here, artists are asked to communicate science in more interesting, approachable, or aesthetically pleasing ways. They are also often delegated responsibility for asking critical or ethical questions, in a way that increases the apparent accountability of scientific institutions whilst keeping these debates at arm’s length. The logic of innovation is mostly associated with industry where, e.g. IT companies have involved artists in product development (Born and Berry [6]). However, we were primarily interested in what Born and Berry term the logic of ontology. The aim here is to find collaborative structures that are more equal—or at least equally awkward—ideally leading to transfers of knowledge and practices across disciplinary boundaries. Artists are not merely involved as an instrument of science, but rather as an equal collaborator with alternative perspectives and approaches. According to Born and Berry this has the potential to change our understanding of science and of the world itself, by offering encounters with multiple alternative ontologies. This experience of different ways of thinking about what kinds of things make up the world can provide a framework for a more open-ended interaction between science and the public.

Artists working with microbes and the problems of microbial entanglement have been growing in numbers in the past decades, alongside the growth of both scientific and cultural engagement with microbes as something other than our enemies. Our engagement with microbes could be argued to be shifting from a dominant narrative of control to one of promise (Paxson and Helmreich [10]). Artists are increasingly using microbes as media and as tools to construct artistic conceptions of a complex, ecosystemic nature in which the boundaries demarcating animals, plants, humans, and a swarming, lively microbial biosphere are continuously breached (Hauser [11]).

Including such artistic interventions into microbiome research into *Mind the Gut* was an easy choice for us, as it aligned with the experimental nature of the exhibition, as well as the more open-ended approach to science communication founded partly on the logic of ontology as outlined above. We included a number of such art works and collaborations. One such example is the work of the artist Kathy High, whose practice lies at the intersection of art, science, and the personal, engaging both ethical dilemmas, speculative futures and existential concerns of biomedicine and biotechnology. We featured three objects from her work around fecal matter transplants (FMT), which she has a personal interest in because of her personal experience of having Crohn’s disease. The first object is a speculative prototype of a DIY stool bank, consisting of a glass container filled with honey and ceramic excrement. The piece belongs to a series ‘The Bank of Abject Objects’ which responds to the notion that healthy feces might become a valuable commodity in the near future. As our internal microbiomes continue to become more unbalanced, mirroring the shifts in our larger ecological sphere, feces might transform from a dangerous waste product that must be cleansed and made invisible into a possible source of ecological intervention.

While High was investigating FMT, a friend asked whose stool *she* would want to use—in a sense, asking which other person she might want to take in. She settled on David Bowie, being a lifelong fan, and decided to make a series of photos of herself costumed as famous images of him. She sent the photographs to Bowie along with a letter asking for an unusual exchange: Whether he would send some of his feces in return. The exchange never happened, as Bowie unbeknownst to the artist was battling cancer at the time. In our exhibition, we display both the letter and one of the images of *Kathy as Bowie* (see Figure 3). These works bind together patient perspectives—the hopes, fears, frustrations, and anxieties connected to suffering from a chronic medical condition that the medical establishment is still trying to figure out—with an artistic, playful reflection upon our entanglement in the microbial biosphere inside and outside of us. Like other potential transplant recipients, the artist wonders what exactly is being exchanged when organic matter is moved from one body to another, something that is further complicated by the possibility of the commensal microbes possibly impacting the mental state and moods of the recipient. High’s work also invites us to ask broader cultural questions of FMT, such as how we might rethink our relationship to feces, why we often are ashamed of our bodily functions, and where this shame came from culturally and historically.

A second collaboration was made with the Canadian scientist and bioartist Francois-Joseph Lapointe. Lapointe is professor of evolutionary ecology at the University of Montreal, and also holds a PhD in dance. His artistic practice revolves around what he terms performance experiments, in which he modifies and studies his own microbes in different ways, using his body as a laboratory and a seismic register of how our microbial constituents shifts through different actions and environments. In *Mind the Gut*, we featured his project *Becoming Batman*, undertaken during a research trip to New Guinea in 2016. While studying the local bat population, Lapointe noticed the locals eating the bats, and decided that he would do the same (along with other local species), while sampling his oral microbiome before and after the meal, to see how consuming the animal changed his microbial population, and in turn, himself. From the data produced by this and similar performance experiments, Lapointe produces what he calls ‘microbiome selfies’, artistically modified data visualizations. He has conducted a number of such selfie projects, including the project *1000 Handshakes* which Lapointe conducted in collaboration with Medical Museion in 2014, where he visited the medical faculty at the University of Copenhagen and shook a 1000 people’s hands; his palm microbiome was then sampled after every 50 handshakes, to see how the contact with other people changed him. The selfies were then exhibited at the Medical Museion in an exhibition entitled *Hello Bacteria!*

Alongside *Becoming Batman*, we also collaborated with Lapointe to produce a series of microbial portraits of a family living in the same house in Montreal, consisting of a baby, a young child, a mother, and a grandmother. Fecal samples from the four family members were collected and sequenced by Lapointe and his team, and then visualized as four slowly turning microbial ‘planets’, whose networked surfaces consisted of dots representing microbial species, and the relative size of the dots the abundance of the given species. The planets show how microbial diversity shifts over the lifetime of the organism: the baby’s microbiome is the least diverse, and diversity then increases through the young child and the mother, and then decreases again in the grandmother. The microbial planets provide a useful talking point for our guided tours as well as an artistic representation of the complicated and interwoven nature of our microbial relations. They communicate directly and easily, but also prompt discussion amongst scientifically trained visitors in how the visualizations were possible and what rhetorical functions they play.

Engaging with artists in the making of the exhibition is a way to highlight how microbes, both in scientific and artistic fields, increasingly are becoming ‘model organisms’, that is, organisms that are made to signify larger biological worlds and imagined futures. More specifically, we have been interested in microbiomes being used as ‘model ecologies’, that is, models for thinking about coexistence and human and nonhuman entanglement (Ankeny and Leonelli [12]). In this light, the work of Kathy High emphasizes a new mode of ecological thinking about intervention, where fecal transplants is both a medical and an existential procedure, transferring qualities from one collective to another. And similarly, the performance experiments of Lapointe points to ecosystemic entanglement in everyday actions, from shaking hands to eating. His artistic practice thus highlights the implications of bacteria as model ecologies, pointing tensions about complexity, reductionism versus holism, and of scales and possibilities of intervention.

## Conclusion

*Mind the Gut* deals with a topic that is public, and which belong to culture and society in several ways: Both because it speaks to fundamental somatic aspects of what it means to be human, and because scientific developments are being reported and brought to the public as they happen, long before basic scientific issues are settled. This makes GBM research both vital and vulnerable to overinterpretation. As microbiome interventions are relatively cheap and accessible, it is also a potentially creative but undisciplined source of personal understanding and treatment, and a potential ‘wild west’ for commercial interests. *Mind the Gut* is a public space that aimed to encourage reflection and curiosity, by showing how biomedicine fits into social, cultural, historical, and directly personal contexts. The exhibition does not aim to provide answers about what food the visitors should eat or what the truth of how gut and brain interactions might be. We emphasize process over result, hopefully encouraging the visitors to ask their own questions of the relationship between mind and gut, between body and microbes.

The exhibition ultimately rests on a series of existential and philosophical questions that have piqued our interest over the last years, but which are only just beginning to be explored. What are we to make of microbial entanglements creeping into the traditional confines of the ‘human experience’? What might it be taken to imply for our self-understanding, both individually and as a community? What sort of social practices and cultural patterns emerge if we see bacteria as foundational to our humanity, and what does it mean for technological and scientific intervention in our distributed environment? And what are the implications for how we philosophically define the human subject, as a thinking, conscious being?

*Mind the Gut* offers no answer to these questions, but rather aims at the more modest goal of starting a conversation about them, the first step in broaching what is likely one of the biggest set of questions in the coming decades: What does it mean to both be and be a part of an ecosystem? How might such questions shift the complex boundaries between mind, body, and environment? Whatever the answers to these questions might become, our work with *Mind the Gut* has emphasized the need for interdisciplinary engagement across art and science.
